# Thoracic stomach syndrome after whole-stomach esophagectomy for esophageal cancer mimicking tension pneumothorax: a case report

**DOI:** 10.1186/s13256-019-2251-0

**Published:** 2019-11-01

**Authors:** Daisuke Hasegawa, Hidefumi Komura, Ken Katsuta, Takahiro Kawaji, Osamu Nishida

**Affiliations:** Department of Anaesthesiology and Critical Care Medicine, Fujita Health University School of Medicine, 1-98, Dengakugakubo, Kutsukake-cho, Toyoake, Aichi 470-1192 Japan

**Keywords:** Thoracic stomach syndrome, Esophageal cancer, Whole-stomach reconstruction, Tension pneumothorax mimic

## Abstract

**Background:**

Sudden onset of respiratory failure is one of the most fearful manifestations in intensive care units. Among the differential diagnoses of respiratory failure, tension pneumothorax is a life-threatening disease that requires immediate invasive intervention to drain the air from the thoracic cavity. However, other etiologies with manifestations similar to those of tension pneumothorax should also be considered after whole-stomach esophagectomy for esophageal cancer. We report a rare case of a patient with thoracic stomach syndrome mimicking tension pneumothorax after esophagectomy with whole-stomach reconstruction.

**Case presentation:**

A 49-year-old Asian woman was admitted to our intensive care unit after esophagectomy for esophageal cancer with whole-stomach reconstruction while under sedation and intubated. Despite initial stable vital signs, the patient rapidly developed tachypnea, low blood pressure, and low oxygen saturation. Chest radiography revealed a mediastinal shift and led to a presumptive diagnosis of tension pneumothorax. Hence, an aspiration catheter was inserted into the right pleural space. However, her clinical symptoms did not improve. Chest computed tomography was performed, which revealed a significantly distended reconstructed stomach that was compressing the nearby lung parenchyma. Her respiration improved immediately after nasogastric tube placement. After the procedure, we successfully extubated the patient.

**Conclusions:**

Similar to tension pneumothorax, thoracic stomach syndrome requires immediate drainage of air from the thoracic cavity. However, unlike tension pneumothorax, this condition requires nasogastric tube insertion, which is the only way to safely remove the accumulated air and avoid possible complications that could occur due to percutaneous drainage. For patient safety, it might be clinically important to place nasogastric tubes after esophagectomy with whole-stomach reconstruction, even if radiographic guidance is required. In addition, clinicians should consider thoracic stomach syndrome as one of the differential diagnoses of respiratory failure after whole-stomach esophagectomy.

## Background

Thoracic stomach syndrome (TSS) is a complication that can occur after esophagectomy for esophageal cancer with whole-stomach reconstruction [1], the main symptom of which is chest discomfort after eating [2]. To our knowledge, our study is the first to describe a severe form of TSS causing life-threatening respiratory failure after esophagectomy with whole-stomach reconstruction. TSS can mimic tension pneumothorax in terms of clinical presentation and chest radiographic appearance, but management of these two conditions is different, making it essential to correctly diagnose TSS. We describe a case of a patient with TSS in whom TSS caused respiratory failure at the time of weaning from mechanical ventilation after esophagectomy for esophageal cancer with whole-stomach reconstruction. This case is unique because it is the first reported case of TSS after esophagectomy with whole-stomach reconstruction that caused life-threatening respiratory failure. In this case, TSS caused respiratory failure in a manner similar to tension pneumothorax through air accumulation in the thoracic cavity and mediastinal shift, and it was successfully diagnosed with chest radiography and computed tomography.

## Case presentation

A 49-year-old Asian woman was admitted to our intensive care unit (ICU) after undergoing esophagectomy for esophageal cancer with whole-stomach reconstruction. She had a past medical history of asthma and cervical/lumbar disk herniation and a surgical history of two cesarean sections and vocal polyp resection under general anesthesia. She had no significant allergies, family history, or environmental or employment history. She was well maintained on 120 mg of fexofenadine hydrochloride and had an inhaler of procaterol hydrochloride and fluticasone furoate/vilanterol trifenatate for her asthma. She has smoked one pack of cigarettes each day for the past 24 years and does not consume alcohol. During the surgery, the surgeons decided not to place a nasogastric tube, because placement of the nasogastric tube was difficult, and they instead tried to avoid leakage at the anastomosis site. After admission to the ICU and sedation, the patient was orally intubated with an endotracheal tube, allowing suction above the cuff (internal diameter 7.0-mm Mallinckrodt™ TaperGuard Evac oral tracheal tube; Covidien, Minneapolis, MN, USA), because we routinely perform extubation and examine the function of the vocal cords on postoperative day 1. The patient’s vital signs were initially stable. Upon physical examination, she was not in acute distress, and she had a heart rate of 82 beats/minute, blood pressure of 110/42 mmHg, respiratory rate of 17 breaths/minute, body temperature of 37.4 °C, and oxygen saturation of 100% with mechanical ventilation (fraction of inspired oxygen [FiO_2_], 0.3) with spontaneous breathing in the pressure support mode. The rest of her physical examination was unremarkable, including the neurological examination. Laboratory studies revealed a white blood cell count of 7300/μl, hemoglobin level of 12.4 g/dl, platelet count of 186 × 10^3^/μl, and hematocrit of 35.1%. The patient’s level of aspartate aminotransferase was 142 U/L, and her alanine aminotransferase level was 153 U/L. Her creatinine level was 0.51 mg/dl. The result of her initial chest x-ray examination in the supine position was unremarkable.

**Fig. 1:**
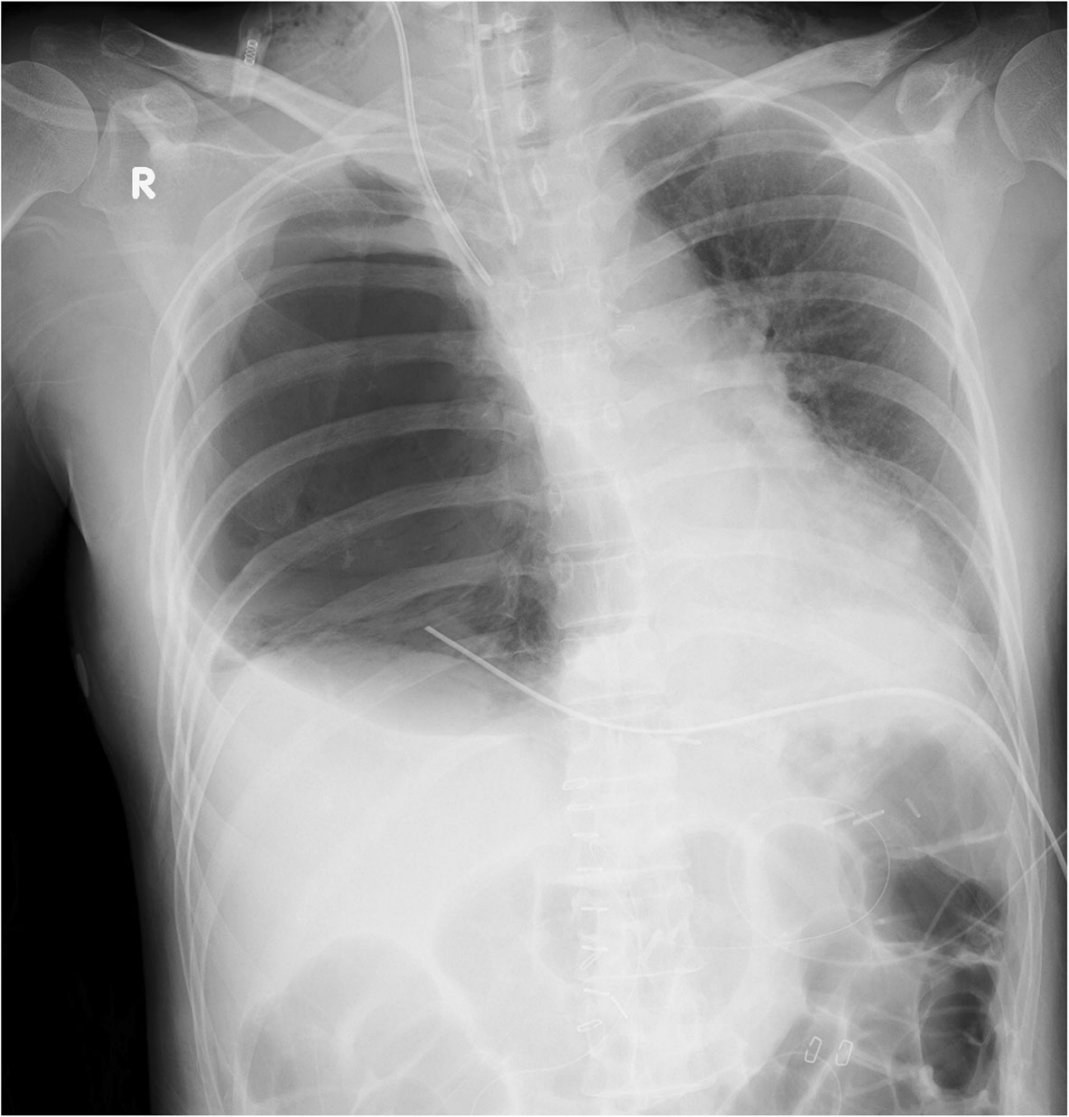
Chest radiograph obtained upon rapid development of tachypnea

**Fig. 2:**
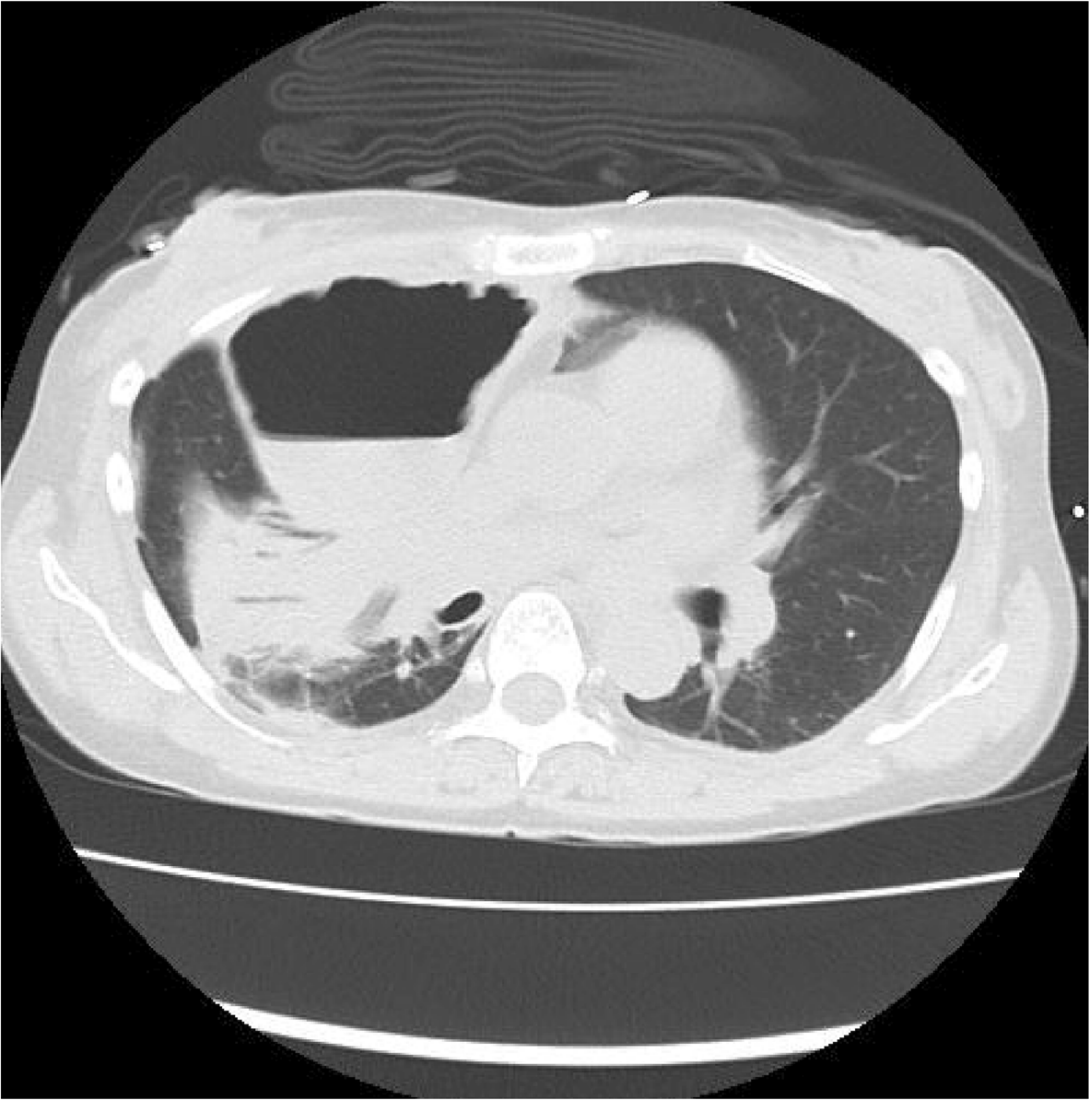
Chest computed tomographic image showing transverse section of the distended, reconstructed whole stomach compressing the proximal lung parenchyma

**Fig. 3:**
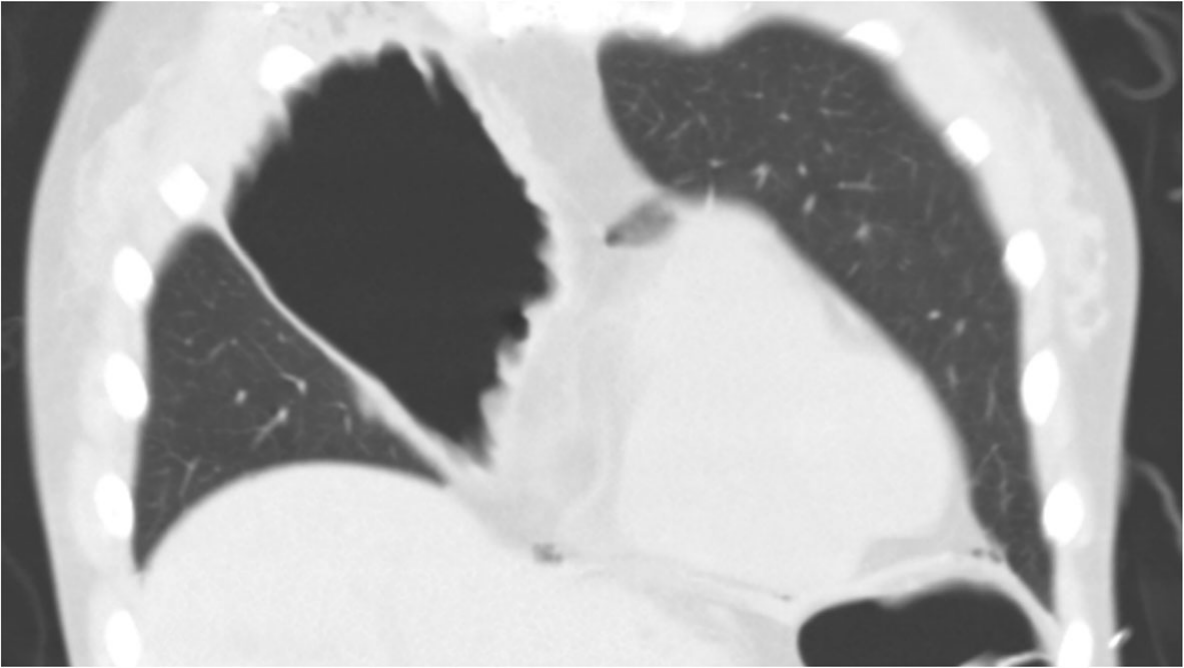
Chest computed tomographic image showing coronal section of the distended, reconstructed whole stomach compressing the proximal lung parenchyma

However, 12 hours after admission to the ICU, the patient rapidly developed tachypnea. On physical examination, she was in acute distress; her heart rate was 78 beats/minute, blood pressure was 92/58 mmHg, respiratory rate was 28 breaths/minute, and oxygen saturation was 96% with mechanical ventilation (FiO_2_, 1.0). Furthermore, chest examination revealed decreased respiratory sounds on the right side. Arterial blood gas analysis revealed that her carbon dioxide pressure was 44 mmHg and oxygen pressure was 109 mmHg, meaning the ratio of oxygen pressure to fraction of inspired oxygen (P/F) was 109 mmHg, together indicating failure of oxygenation. Chest radiography performed with the patient in the supine position revealed a mediastinal shift without lung parenchymal markings in the upper and middle lung fields (Fig. 1). Chest ultrasonography revealed absence of the sliding sign. Hence, an aspiration catheter was inserted into the right pleural space under a presumptive diagnosis of tension pneumothorax 2 hours after the development of respiratory distress. Dark red blood (300 ml) and a small amount of air were released from the catheter, and the P/F ratio improved slightly to 236 mmHg. Subsequently, chest computed tomography was performed, which revealed a significantly distended reconstructed stomach that was compressing the nearby lung parenchyma (Figs. 2 and 3). The patient’s respiration improved markedly immediately after portable X-ray fluoroscopy-guided nasogastric tube placement in the ICU 5 hours after the aspiration catheter insertion. Chest radiography performed after nasogastric tube placement revealed return of the mediastinum to the right, indicating right lung inflation. The patient’s respiratory rate decreased to 15 breaths/minute, and her P/F ratio improved to 516 mmHg. After the procedure, we were able to successfully extubate the patient 30 minutes after nasogastric tube placement. On the 3rd day after admission to the ICU, the patient was moved to the medical ward, and on the 18th day after admission to the ICU, she was discharged to home without any problems. At a hospital visit 1 year after discharge, the patient claimed that she was doing well, and though she occasionally experienced chest discomfort after eating, she had not experienced severe dyspnea or respiratory problems since her stay in the ICU.

## Discussion

We describe the first reported case of TSS after esophagectomy with whole-stomach reconstruction in which TSS caused life-threatening air accumulation in the thoracic cavity and mediastinal shift mimicking tension pneumothorax and was diagnosed with clinical symptoms, chest X-ray imaging, and computed tomography. TSS after surgery [1] is caused by an enlarged stomach compressing the lungs and mediastinum on the surgical side [2]. To our knowledge, the incidence of TSS is as high as 5.3% after whole-stomach esophagectomy [3]. TSS is clinically diagnosed, and its severity is measured qualitatively by clinical symptoms. Aside from a surgical manifestation, this syndrome can also occur after overeating and can be eased by fasting or vomiting [4].

However, clinical presentations of TSS reported in previous literature have never been severe enough to require immediate intervention. There are some reports on fatal TSS or tension gastrothorax that mimicked tension pneumothorax, similar to our patient’s case, caused by congenital diaphragmatic hernia or traumatic diaphragmatic injury [5, 6]. However, to our knowledge, the present case is the first reported case of TSS after esophagectomy with whole-stomach reconstruction. We suspect that this case occurred because of the aerophagia caused by positive pressure mechanical ventilation. The abnormal level of air accumulation was likely caused by the entry of air not into the lungs but into the reconstructed stomach. Thus, nasogastric tube placement is the most effective way to drain the air in the reconstructed stomach to prevent this complication, especially in patients on mechanical ventilation.

Nasogastric tube placement has been one of the most controversial issues in the perioperative care of esophagectomy, and recent studies claim that nasogastric tube omission would be safer, as observed for other gastrointestinal surgeries [7]. Theoretically, the anastomosis site should be susceptible to the poke or suction of a nasogastric tube, thus increasing the risk of anastomosis leak. However, one randomized controlled trial with 40 patients found that the risk of anastomosis leak was significantly higher in the routine nasogastric tube group than in the nasogastric tube omission group [8]. However, it is notable that this study was done in a single institution and with a relatively small sample size [8]. Thus, further research is required to demonstrate that anastomosis leak occurs more often in patients with nasogastric tubes after esophagectomy, and prevention of fatal TSS complication by nasogastric tube placement should still be considered an important treatment option.

Additionally, TSS should be considered when clinicians encounter patients with respiratory failure after esophagectomy with whole-stomach reconstruction, because management of this condition differs from that of tension pneumothorax. Sometimes, performing nasogastric tube placement can be difficult because of the nonphysiological shape of the stomach after reconstruction. However, this procedure should be performed under radiographic guidance to avoid this fatal complication. In this case, on the basis of the nature of the aspirated fluid, we suspect that we punctured the gastric wall from the outside. The procedural approach in our patient’s case would have differed had we known that TSS would be one of the differential diagnoses of respiratory failure after whole-stomach esophagectomy.

## Conclusions

Physicians should be aware that TSS may be a complication after esophagectomy with whole-stomach reconstruction and that its primary presentation can be respiratory failure. Additionally, performing prophylactic nasogastric tube placement to avoid TSS is important to ensure patient safety and improve overall outcomes, especially for patients on mechanical ventilation.

## Data Availability

Data sharing is not applicable to this article, because no datasets were generated or analyzed in the present case report.

## Abbreviations

FiO_2_: Fraction of inspired oxygen
ICU: Intensive care unit
P/F: Ratio of oxygen pressure to fraction of inspired oxygen
TSS: Thoracic stomach syndrome
